# From ideation to attempt: A study of suicidality and its correlates amongst patients with schizophrenia in a resource-poor country

**DOI:** 10.4102/sajpsychiatry.v28i0.1547

**Published:** 2022-01-27

**Authors:** Oluseun P. Ogunnubi, Francis O. Aina, Cecilia O. Busari, Olamijulo Fatiregun, Babatunde Fadipe, Abosede A. Adegbohun, Osunwale D. Oni

**Affiliations:** 1 Department of Psychiatry, Faculty of Clinical Sciences, College of Medicine, University of Lagos, Lagos, Nigeria; 2 Department of Psychiatry, Faculty of Clinical Services, Lagos University Teaching Hospital, Lagos, Nigeria; 3 Department of Psychiatry, Faculty of Clinical Services, Federal Neuropsychiatric Hospital, Lagos, Nigeria

**Keywords:** schizophrenia, suicidal behaviours, suicidal ideation, suicidal plan, suicidal attempt

## Abstract

**Background:**

There is increasing evidence that patients with schizophrenia have high tendency to commit suicide. However, such an act is usually preceded by suicidal behaviours (or suicidality) such as suicidal ideations, suicidal intent, suicidal plans and suicidal attempt. If any of this suicidal behaviour spectrum is missed, then suicide results. In spite of the relevance of such behaviours in the management and prognosis of schizophrenia, there is a paucity of research on the patterns and correlates of suicidal behaviours amongst this population group in sub-Saharan Africa.

**Aim:**

This study assessed the prevalence and pattern of suicidality and its relationship with certain sociodemographic and clinical variables.

**Setting:**

The study was conducted at the outpatient psychiatric clinic of the Lagos University Teaching Hospital, Idi-Araba, Lagos State.

**Methods:**

Suicidal behaviours were assessed amongst 160 randomly selected patients with schizophrenia over a six-month period. The prevalence, pattern and correlates of suicidal behaviour amongst them were also assessed. Data were collated and processed with the eighteenth version of Statistical Package for Social Sciences (SPSS 18).

**Results:**

About 43.1% of the participants exhibited suicidal behaviour. Of these, 5.8% exhibited suicidal plans whilst 75.4% attempted suicide. In terms of severity, one tenth (10%) of those who expressed suicidal behaviour exhibited severe suicidal tendencies. Participants who earned lesser income were more likely to exhibit suicidal behaviour. The same pattern was observed for medication adherence viz a viz suicidal behaviour.

**Conclusions:**

The study brings to the fore the tendency of patients with schizophrenia to commit suicide, hence the need to screen for suicidal behaviour before suicide eventually occurs.

## Introduction

Suicidality or suicidal behaviour can be expressed either as suicidal ideation, suicidal intent, suicidal plan or suicidal attempt.^1,2^ Suicidal ideation, intent, planning and attempt are precursors to suicide. Hence early identification and assessment of any of these is critical.^3,4^

Suicide amongst patients with schizophrenia has been a significant area of concern in psychiatry. For example, a large 5-year World Health Organization study which comprised of 1056 patients who exhibited psychotic symptoms found that suicide was the most common cause of death amongst these patients.^5^

Likewise, Meltzer and colleagues in their study found that approximately 50% of patients with schizophrenia or schizoaffective disorder attempted suicide, whilst almost 10% of them died of suicide.^1^

Factors such as unemployment,^6,7^ single marital status^7^ and solitary living^8^ have been found more often in patients with schizophrenia who attempted and completed suicide. Patients with schizophrenia who exhibit suicidal behaviour tend to be admitted twice as much as those who do not, although it is unclear whether other factors are contributory.^8^

There is a dearth of study on the prevalence of suicidal behaviour amongst patients with schizophrenia in Nigeria. However, a study by Gureje et al. in 2007 found a lifetime prevalent estimates of suicidal ideation, plan and attempts to be 3.2% (95% CI 1.4–6.5), 1.0% (95% CI 0.4–7.5) and 0.7% (95% CI 0.5–1.0) respectively.^9^ The rationale for this study is thus to study the rate of suicidal behaviour amongst patients with schizophrenia.

Unfortunately, psychiatrists often tend to focus on the psychopathology and the biological management of patients with schizophrenia without paying adequate attention to the suicidal behaviour in these patients.^1^ This may then explain why research has shown that patients who eventually committed suicide had previously talked about it either directly or indirectly with their psychiatrists.^1^ This study further aimed at studying the pattern of suicidal behaviour as well as the associated risk factors of suicidal behaviour amongst patients with schizophrenia. It is of note that, watching out for suicidal behaviour with prompt intervention is a good suicide prevention strategy.^10^

## Methods

The study population comprised of patients with schizophrenia who attended the psychiatry outpatients’ clinic of the Lagos University Teaching Hospital. It is a federal government owned tertiary health institution located in Nigeria. The total registered outpatients with a diagnosis of schizophrenia was 400.^11^ It was a descriptive cross-sectional study. A sample size of 160 participants was arrived at, and these were selected from the cohort using a systematic random sampling method.

### Sample size determination

In a Finnish sample, 45 (51%) of 89 schizophrenics who committed suicide had their last contact with a health care provider within four days of death, 70% within two weeks, and 82% within a month.^12^ Because the suicidality module of MINI 6 used in this study assesses both ‘within a month’ and ‘a life time risk’ of suicidal behaviour, an average prevalence of 82% was used as a guide to calculate the sample size.

Thus, using the formular by Araoye:

*n* = [z^2^p (1-p)/d^2^]^13^

Thus (*n*) = 3.84 x 0.82 (1–0.82) / 0.0025 which equates to 230.

### Inclusion criteria

1. Adults aged 18 years and above.
2. Prior diagnosis of schizophrenia from the case notes.
3. Those confirmed as having schizophrenia.
4. The patients included in the study were those who had been coming to the clinic and had not relapsed in the preceding one month.
5. Patients who gave consent to participate in the study.

### Exclusion criteria

1. Patients who are not literate enough to read and write in English with regard to completing the questionnaires.
2. New patients who were attending the clinic for the first time for investigations and assessment.
3. Acutely ill patients that were adjudged by the researcher to be too ill to communicate effectively.

### Study instruments

#### Socio-demographic and Clinical Questionnaire (SDQ)

This was designed by the authors for the purpose of the research. It comprised of four sections.

**Section A:**

- Age, sex, marital status, and educational level amongst others.
- Earnings/income per month, amount spent on treatment per month, any additional financial support and the kind of house lived in.

**Section B:**

- Medication side effects and adherence (using the Modified Morisky Medication Adherence Scale). The scale was successful in predicting positive therapeutic outcomes and has been validated and used in Nigeria.^14^

**Section C:**

- Alcohol/psychoactive substance use.

**Section D:**

- Illness-related variables.

#### MINI International Neuropsychiatric Interview (MINI 6)

The English version 6.0.0 was used by the researchers to confirm the diagnosis of schizophrenia and to determine the presence or absence of suicidality. MINI 6 is a structured diagnostic interview.^15^ It was developed to assess the diagnoses of psychiatric patients according to DSM-IV and ICD-10 criteria.^15^ A score of 1–8 points is low; 9–16 points is moderate and >17 points is high on the suicidality scale module of MINI International Neuropsychiatric Interview (MINI 6).^15^ It is less time consuming than the Schedules for Clinical Assessment in Neuropsychiatry (SCAN), that is, 18.7 ± 11.6 min, median 15 min than the above referenced instrument. This instrument had been used in some Nigerian studies.^16,17^

### Study procedure

The ‘psychotic module’ of the MINI 6 was administered to confirm the diagnosis of schizophrenia. The participants who met the inclusion criteria were then given the Sociodemographic and Clinical Questionnaire (SDQ) which included questions on age, gender, marital status, and educational level, amongst others. Each booklet was serialised, and the patients were given corresponding tally numbers. The English version 6.0.0 of MINI was also used by the researchers to determine the presence or absence of suicidality. The MINI diagnostic instrument is divided into different modules identified by letters corresponding to diagnostic categories. Each module screens for various psychiatric conditions such as major depression, dysthymia, suicidality, hypomania, and agoraphobia, to mention but a few. The ‘suicidality module’ of the MINI 6 was administered by the researchers to assess the respondents’ current and lifetime suicidal behaviour. Some examples of the questions in MINI are as follows: In the past month, did you suffer an accident? Did you plan or intend to hurt yourself in that accident, either actively or passively?

The respondents were expected to answer either ‘yes’ or ‘no’. The instrument has different allotted points for each ‘yes’ answered. The C1–C8 questions of MINI diagnostic instrument assess for the current risk of suicide whilst the last question – QC9 assesses for the lifetime risk. In our study, we used both the current suicide risk (QC1–QC8 of MINI) to calculate the total scores for the frequency and severity of suicidality amongst the participants. In contrast, the lifetime risk scores (QC9 of MINI) were used to determine the frequency of suicidal attempt alone. All questions were rated. A score of 1–8 points is low; 9–16 points is moderate, and >17 points is high on the suicidality scale module of MINI International Neuropsychiatric Interview (M.I.N.I 6).^15^

### Data collection procedure

The lead researcher liaised with the medical record officer on the clinic day, selected the case files of all patients with recorded diagnosis of schizophrenia that were scheduled for appointment on each particular clinic day. The first person was selected by simple random sampling after which others were selected by systematic random sampling method. The maximum number that could be interviewed was 10 out of an average total attendance of 30 patients with the diagnosis of schizophrenia for each clinic day. Hence, the sampling interval rate of 3 was used for the study.

In a situation where a subject did not meet the selection criteria, the next subject meeting the selection criteria was recruited. This continued until the total number of calculated sample size was completed. The interview session took an average of 30 min.

The lead researcher who was a psychiatrist trainee was trained by a consultant psychiatrist (O.A.) who had also used it for research purposes. There was no need for a research assistant.

The data collection took eight months to be completed.

### Ethical considerations

Ethical approval was obtained from the Health Research Ethics Committee of the LUTH. Written consent was obtained from each participant following a detailed explanation about the aims and objectives of the study. In those found to be positive for suicidality, the implications were explained to them, and they were appropriately referred to their attending Psychiatrists for review. Those requiring additional assistance, like the need for continuous cognitive behavioural therapy were also referred to the clinical psychologists.

### Data analysis

Data were analysed using Statistical Package for Social Sciences (SPSS), version 18. Comparison between categorised sociodemographic/clinical variables and suicidality was made using the chi-square test. The description of the pattern of suicidality amongst participants was done by categorising them using the most severe form of suicidal behaviour in each respondent. In a situation where a participant had more than one suicidal event, for example, *if a participant had a suicidal intention and suicidal attempt, the most severe form of suicidality in that continuum being suicidal attempt was chosen for that particular participant*.

The non-parametric equivalent of the inferential statistics tests was used as appropriate: Mann-Whitney *U* test instead of independent *t*-test and Spearman’s correlation was used instead of Pearson correlation.

In order to determine the variables that were independently associated with suicidality in the patients with schizophrenia, all the variables that were associated with suicidality in the previous analysis were re-entered into binary logistic regression analysis using suicidality as the dependent variable.

## Results

### Sociodemographic and clinical characteristics of participants

The participants were aged between 20 and 71 years, with a mean age of 38.54 (±11.30) years (Table 1). Slightly more than half of the participants (50.6%) belonged to the 26–40 years age group. More than half of the participants were females (53.8%), single (51.9%) and employed (59.4%).

**TABLE 1:** Socio-demographic data of participants (*n* = 160).

Variable	Frequency	Percentage
**Age (years)**		
18–25 (Early adult)	18	11.2
26–40 (Adult)	81	50.6
41–60 (Late adult)	53	33.2
> 60 (Geriatric)	8	5.0
**Gender**		
Male	74	46.2
Female	86	53.8
**Marital status**		
Single	83	51.9
Married	62	38.8
Divorced	8	5.0
Separated	2	1.2
Widowed	5	3.1
**Formal educational status**		
Nil	2	1.2
Primary	10	6.2
Secondary	42	26.3
Tertiary	106	66.3
**Employment status**		
Employed	95	59.4
Unemployed	65	40.6
**Tribe**		
Yoruba	112	70.0
Ibo	34	21.2
Other	14	8.8
**Income (*N*)**		
None	35	21.9
1 – < 18 000	36	22.5
18 000 – < 36 000	36	22.5
36 000 – < 54 000	10	6.3
54 000 – < 72 000	14	8.8
≥ 72 000	29	18.1
**Amount spent on illness monthly (*N*)**		
< 5000	73	45.6
5000 – < 10 000	39	24.4
10 000 – < 15 000	30	18.8
≥ 15 000	18	11.2
**Additional financial support**		
No	70	43.7
Yes	90	56.3
**Age at onset of illness (years)**		
< 18 (Adolescence)	6	3.8
18 – 25 (Early adult)	69	43.1
26 – 40 (Adult)	70	43.8
41 – 60 (Late adult)	13	8.1
> 60 (Geriatric)	2	1.2
**Duration of illness (years)**		
1–7	84	52.5
8–15	44	27.5
> 15	32	20.0
**Number of episodes of relapse**		
1–3	108	67.5
4–6	43	26.9
≥ 7	9	5.6
**Number of admissions**		
Nil	17	10.6
1–3	114	71.3
≥ 4	29	18.1
**Co-morbid chronic physical illness**		
Yes	13	8.1
No	147	91.9
**Duration on antipsychotics (Years)**		
1–7	85	53.1
8–15	50	31.3
≥ 16	25	15.6

Mean age = 38.54 (±11.30) years; median age = 38 years.

Less than half (43.8%) of the participants had their first episode of the illness at age 26–40 years (Table 2). Nearly seven out of 10 participants (67.5%) have had at least three episodes of schizophrenia. Likewise, about seven out of 10 participants (71.3%) have had three hospital admissions on account of the disorder.

**TABLE 2:** Frequency and severity of suicidality amongst the participants (*n* = 160).

Variable	Frequency	Percentage
**Presence/absence of suicidality**		
No suicidality	91	56.9
Suicidality	69	43.1
**Severity of suicidality (*n* = 69)**		
1–8 (mild suicidality)	45	65.2
9–16 (moderate suicidality)	7	10.1
> 17 (severe suicidality)	17	24.7
**Pattern of the maximum expression suicidality as expressed by the participants (*n* = 69)**		
Suicidal ideation	5	7.2
Suicidal intent	8	11.6
Suicidal plan	4	5.8
Suicidal attempt	52	75.4

Note: The description of the pattern of suicidality amongst participants was done by categorising them using the most severe form of suicidal behaviour, especially where participants had more than one suicidal event.

### Frequency and severity of suicidality amongst the participants

In general, 69 (43.1%) participants had suicidal behaviour out of which 17 (24.7%) had severe suicidality, 7 (10.1%) had moderate suicidality whilst 45 (65.2%) had mild suicidality.

Of the 69 participants that met the criteria for suicidality, majority (75.4%) was assessed to have had suicidal attempt (Table 2).

### Relationship between sociodemographic and clinical attributes of the participants and suicidality

The mean age of the participants who had suicidality was 36.75 (±10.54) years in comparison with the participants without suicidality who had a mean age of 39.89 (±11.7) years (Table 3). There was no significant statistical difference between the ages of participants who had suicidality and those who had none (*t* = –1.750, *p* = 0.082). The proportion of participants with suicidality was almost equally distributed within the gender with male and female being 43.2% and 43.0% respectively.

On the contrary, whilst Table 2 showed the percentage distribution of the income earned by the patients, Table 3 showed a significant statistical association between income earned per month as well as income spent per month on the illness and suicidality. This means, the less a participant earned in a month, the more he or she was likely to exhibit suicidal behaviour. The same applies to the amount participants spent monthly on their health. The more they spend on their health monthly, the more they are likely to exhibit suicidal behaviour (*U* = 2374.000, *p* = 0.008) and (*U* = 2374.000, *p* = 0.008) respectively.

**TABLE 3:** Relationship between socio-demographic and clinical attributes of participants and suicidality (*n* = 160).

Variable	Suicidality			No suicidality			Statistics		
	*N* = 69			*N* = 91					
	*n*	%	Mean ± SD	*n*	%	Mean ± SD			
**Age** (in years)	-	-	36.75 ± 10.54	-	-	39.89 ± 11.7	*t* = -1.750	*df* = 158	*p* = 0.082
**Gender**									
Male	32	43.2	-	42	56.8	-	*X^2^* = 0.001	*df* = 1	*p* = 0.978
Female	37	43.0	-	49	57.0	-			
**Marital status**			-			-			
Married	26	41.9	-	36	58.1	-	*X^2^* = 0.058	*df* = 1	*p* = 0.809
Unmarried	43	43.9	-	55	56.1	-			
**Employment status**			-			-			
Employed	37	38.9	-	58	61.1	-	*X^2^* = 2.531	*df* = 1	*p* = 0.197
Unemployed	32	49.2	-	33	50.8	-			
**Formal educational status**			**-**			**-**			
Up to secondary school	28	51.9	-	26	48.1	-	*X^2^* = 2.531	*df* = 1	*p* = 0.112
Above secondary school	41	38.7	-	65	61.3	-			
**Tribe**			-			-			
Yoruba	50	44.6	-	62	55.4	-	*X^2^* = 3.418†	*df* = 1	*p* = 0.167
Ibo	10	30.3	-	23	69.7	-			
Other	8	57.1	-	6	42.9	-			
**Income earned per month (*N*) (n/mean rank)**	69	69.41	-	91	88.91	-	*U* = 2374.000	*z* = -2.654	*p* = 0.008
**Amount spent on illness per month (*N*) (n/mean rank)**	69	69.34	-	91	88.96	-	*U* = 2369.000	*z* = -2.660	*p* = 0.008
**Age at onset of illness (years)**	-	-	27.08 ± 9.17	-	-	29.32 ± 9.53	*t* = -1.491	*df* = 158	*p* = 0.138
**Duration of illness (years)**	-	-	9.69 ± 8.34	-	-	10.36 ± 10.34	*t* = -0.441	*df* = 158	*p* = 0.660
**Number of episodes of relapse**	-	-	3.29 ± 2.13	-	-	3.09 ± 2.05	*t* = -0.606	*df* = 158	*p* = 0.545
**Admitted before?**									
Yes	10	58.8	-	7	41.2	-	*X^2^* = 1.911	*df* = 1	*p* = 0.200
No	59	41.3	-	84	58.7	-	-	-	-
**Co-morbid chronic physical illness**									
Yes	5	38.5	-	8	61.5	-	*X^2^* = 0.125	*df* = 1	*p* = 0.779
No	64	43.5	-	83	56.5	-			
**Duration on antipsychotics (in years)**	-	-	8.53 ± 6.63	-	-	8.74 ± 7.74	*t* = -0.183	*df* = 158	*p* = 0.855

? , Fisher’s Exact Test (used where observed cell value (0) was small and chi square inappropriate).

*U*, Mann-Whitney *U* test (a non-parametric test) used instead of *t* test because the variables were not normally distributed; *t*, independent t-test; *X^2^*, Pearson Chi Square.

Overall, there was no significant statistical relationship between suicidality and all the measured clinical attributes of the participants.

### Logistic regression analysis of the factors independently associated with suicidality amongst the subjects

In order to determine all the variables that were independently associated with suicidality in the subjects, all variables that were associated with suicidality in previous analyses (i.e. income per month and amount spent on care monthly) were entered into binary logistic regression analysis using suicidality as the dependent variables (Table 4). However, none of these two factors was found to predict suicidality amongst the participants independently.

**TABLE 4:** Logistic regression analysis of the factors associated with suicidality amongst the participants (*N* = 160).

Variables	β	SE	Wald	*p*	Odds ratio (Exp B)	95% CI	
						Lower	Upper
Income per month	0.000	0.006	0.002	0.960	1.000	0.988	−1.011
Amount on care per month	−0.064	0.039	2.673	0.102	0.938	0.868	−1.013
Additional support (Nil support)	−0.721	0.466	2.391	0.122	0.486	0.195	−1.213

## Discussion

This study reported the current rates of suicidality amongst patients with schizophrenia. Some earlier research carried out in Nigeria looked at one aspect of suicidality or the other in different groups of people but not suicidality in its entirety. Such studies include suicidal ideation in patients with epilepsy,^16^ and sickle cell disease,^17^ suicidal attempt in a military setting,^18^ suicidal ideation amongst those living with human immunodeficiency virus and/or acquired immune deficiency syndrome (HIV/AIDS),^19^ and suicidal attempt amongst adolescents.^20^

The sociodemographic profile of the respondents showed that their ages ranged from 20 to 71 years with a mean age of 38.54 (±11.30) years and a median age of 38 years. The mean age of the participants in the current study was very close to that reported in some earlier studies conducted in Nigeria^21,22^ Kuwait^23^ and Malasia.^24^ Our study showed a slightly higher proportion of females when compared to other hospital studies on schizophrenia.^22,25^

This study, like many other earlier studies, showed that the majority of the participants were not married. This finding aligns with previous knowledge from the literature that patients with mental illness such as schizophrenia are more likely to be unmarried when compared to other patients without mental disorders.^26,27^ This is because schizophrenia has been said to be a highly stigmatised disorder and having such a diagnosis may significantly reduce the chances of securing a marital relationship amongst people affected with this condition.^28^

Contrary to previous studies by Reavley and Jorm, Adewuya and Makanjuola, and that of Gureje and Bamidele which showed that patients with schizophrenia have poor long term social outcome in maintaining a job due to stigmatisation, which make them manifest a downward drift on the social ladder, over half of our study participants (59.4%) were gainfully employed.^22,26,28,29^ The reason for the difference in the present study with those cited earlier could be because our participants were stable enough to take up one form of employment or the other at the time the study was conducted.

Almost a quarter of the patients (21.9%) did not earn any income at all whilst 56.3% of the subjects had additional financial aid.

About half (43.8%) of the subjects reported that they had their first episode of the illness between the ages 26 and 40 years with mean age of onset put at 28.35 (±9.41) years and median age of 27 years. The median age of onset in our study is consistent with a recent study by Adegbohun at Federal Neuropsychiatric Hospital, Yaba, Lagos, where she found the median age of onset amongst patients with schizophrenia to be 27 years.^30^ However, in contrast to the result of our study on the mean age of onset, Maggio and colleagues in France found a mean age of 20.4 (±3.0) years.^31^ The differences in this mean ages could have been because of a methodological variance. Maggio et al. study was a cohort whilst this current study is a cross-sectional one, which is prone to recall bias.^31^

More than half of the participants (67.5%) in this study have had at least three episodes of illness and 71.3% have also had three hospital admissions on account of schizophrenia. A repeated number of relapses and hospital admission such as these may hamper individuals with a chronic mental health disorder such as schizophrenia from contributing meaningfully to the growth of their nations.^32^

Eight per cent of the patients had co-morbid chronic physical illness. This is similar to what Adewuya and Makanjuola found in their study on the subjective quality of life amongst patients with schizophrenia, where they found that 8.1% of the study participants had co-morbid physical illness.^22^ This is however in contrast with Koranyi study where he estimated that 45% of patients with schizophrenia in California’s public mental health system had physical disease and, of these, 47% were undetected by the treating doctors. A substantial proportion of these illnesses were judged to be either causing or exacerbating the patients’ mental illness.^33^ Similarly, Hall et al. found that 46% of patients with schizophrenia who were admitted into a research ward had an unrecognised physical disease that either caused or exacerbated their psychiatric illness.^34^ Despite being asked directly for these comorbidities, the disparity found in this study could be because of the fact that the participants have not been screened or diagnosed with having comorbidities prior to our study. It could also be because of the fact that the patients with schizophrenia prioritised the mental illness above their physical ailment and as such did not report them during the study.

### Frequency and severity of suicidality

About 6 in 10 study participants had zero scores on current suicidality module of MINI, which indicated an absence of suicidality. In contrast, 43.1% had at least a score and above on the current suicidality risk module of the MINI questionnaire.

The result of this study may not be easily comparable with earlier studies as none of the studies available for review examined the prevalence of suicidality as a whole in patients with schizophrenia. Most of the previous studies focused on either suicidal ideation or completed suicide amongst patients with schizophrenia.

Even though religion and cultural belief in Nigeria are against suicide,^20^ the high frequency of suicidality in this study shows that patients with schizophrenia are still not immune to having suicidal behaviour. The prevalence of suicidality in our study falls within the estimated 18% – 55% current risk of attempted suicide in patients with schizophrenia as found by Kasckow and colleagues in their research on the suicidal behaviour in older patients with schizophrenia.^4,35^ The high frequency of suicidality found in this study might be attributed to the stigma associated with schizophrenia in this part of the world. Depression as an integral part of schizophrenia could be a very prominent factor that might have accounted for the high prevalence of suicidality amongst our study population. Feelings of depression are considered an integral part of suicidal behaviour.^36,37,38.^ More than half of the patients with schizophrenia will experience at least one major depressive episode.^39,40^

Other psychopathology associated with schizophrenia include post schizophrenic depression, obsessive thoughts and hallucination. The presence of command auditory hallucinations (CAH) for suicide alone cannot predict suicidal behaviour, but individuals who are at risk for suicide are more likely to make an attempt when they have CAH.^41^

Poor insight, side effects of medication and poor quality of life amongst people with schizophrenia could be contributory. However, Radomsky et al. found in their study on suicidal behaviour before the first hospitalisation, for example, a lower prevalence of 30.2% lifetime history of suicide attempts amongst all patients with schizophrenia and other psychotic disorders.^42^ The variation of the results of the current study with that of Radomsky et al., which had a lower prevalence, could be because of the difference in the methodology used.^42^ Whilst our study looked at suicidality in its entirety (including ideation, intention, plan, attempt), the study conducted by Radomsky et al. focused on suicidal attempt alone. It is also not impossible that the clinic setting where this current study took place afforded the subjects ample opportunities to express themselves more freely and talk about their suicidal experiences, hence the reason for a higher frequency.^42^

Other social factors which include pre-morbid social functioning, social trauma, and social support may affect the risk of suicidality in patients with schizophrenia. Many investigators suggest that persons with good pre-morbid social functioning who have schizophrenia become demoralised by the decrease in their ability to function and therefore become suicidal. This demoralisation syndrome has been demonstrated in patients who have insight about their illness.^43,44^

Reductions in support – whether temporary, as in therapist or family vacations, or more long term, such as moving out of a family residence – are periods of increased risk.^6^

### Correlates of sociodemographic and clinical attributes of the participants and suicidality

Gender wise, the proportion of subjects with suicidality in this study were almost equally distributed, with male and female being 43.2% and 43.0% respectively. Earlier studies have reported a preponderance of males at risk of suicidal behaviour.^6,45^ The finding of more females who expressed suicidality in our study could have been because of the over-representation of females in our study. Other studies that reported lower risk of suicidal behaviour could have done so because it is assumed that several protective factors from suicide may be at work in women. These include the tendency to seek help when they are depressed, a less intense wish to die when attempting suicide and lower rates of substance use disorders.^46^

Age was not significantly found to be associated with suicidal behaviour in the current study. Kessler et al., in their study on the prevalence and risk factors for lifetime suicide attempts in the National Co-morbidity Survey in 1999, reported that the young are more likely to harbour suicidal ideation and that ages less than 25 years are more strongly related to ideation.^47^ The reason for the disparity could be because of the difference in the instrument used in the methodology.

There was no statistical significance between marital status and suicidality (*p* = 0.809), although some findings have suggested that being married reduces the risk of suicidal behaviour. This may be as a result of enhanced social support received from a primary partner.^48^ The difference in this current study may then suggest that the assessment of the quantity of social contact may be more important than marital status alone for evaluating the risk of suicide in patients with schizophrenia. It has been shown that treatments designed to enhance social networks might have value in reducing the risk for suicidal behaviour amongst patients with psychosis.^42^

Employment status was not found to be significantly associated with suicidality in this current study even though more than half of the group with suicidality were unemployed. Previous studies have, however, in contrast, demonstrated that unemployment is a significant risk factor for suicidal behaviour.^49,50^

Education is presumed to bring about more economic empowerment and good quality of life and as a result reduces other risks of suicide such as poverty, homelessness and unemployment.^47,51^ But this was not the case in our study as educational status was not found to be associated with suicidality (*p* = 0.112).

There was a significant relationship between the income received per month and suicidality as patients who earned more income were less likely to express suicidal behaviour than those earning less. This economic implication of schizophrenia as a chronic illness has been researched extensively.^52^

In the same vein, this study revealed that the more patients spent on their illness monthly, the more they were likely to have suicidality.

There was no significant association between all the measured clinical variables and suicidal behaviour in this study group.

## Conclusion and recommendations

The study brings to the fore not only the tendency of patients with schizophrenia to commit suicide, but also the factors associated with such suicidal behaviour, hence the need to screen for suicidality amongst this population group. If this is strictly adhered to, then suicide can be prevented amongst patients with schizophrenia. Timely referral to a mental health specialist on suicidality for in-depth assessment and establishment of National Suicide Prevention Strategy Centres are amongst the actions that may be taken.

### Limitations

Generalisability of our study to the population will be limited because of bias inherent in tertiary-care based study with potential for more severe cases. This hospital-based study of outpatient clinic attendees may not adequately represent the general population of patients with schizophrenia. However, it provides data for comparison when future studies are conducted on similar sub-population in other mental health-related support service centres. There is a relative absence of local literature on the suicidality in patients with schizophrenia which would have enhanced useful comparison of methodologies and results. Cultural norms, such as moral objections to suicide, could have affected appropriate response to enquiries about suicidality. The researchers did not measure the severity of the psychopathology amongst the patients, so we do not know whether severity of the illness significantly associated with suicidal behaviours. The study also did not look at the prevalence of depressive symptoms in the study population. This would have been appropriate considering the established relationship between depression and suicide.

The strength of the study lies in the fact that international standardised instruments were deployed in all other measured aspects of the study except the sociodemographic and clinical questionnaire. Also, the study was able to subcategorise the suicidality module of MINI, which enabled us to determine the pattern of suicidality amongst the study group.
